# New priorities for climate science and climate economics in the 2020s

**DOI:** 10.1038/s41467-020-16624-8

**Published:** 2020-08-13

**Authors:** David A. Stainforth, Raphael Calel

**Affiliations:** 1 Grantham Research Institute on Climate Change and the Environment, London School of Economics and Political Science, London, UK; 2 Centre for the Analysis of Timeseries, London School of Economics and Political Science, London, UK; 3 Department of Physics, University of Warwick, Coventry, UK; 4 McCourt School of Public Policy, Georgetown University, Washington, DC USA

**Keywords:** Climate change, Climate-change policy, Environmental economics, Developing world, Climate change

## Abstract

Climate science and climate economics are critical sources of expertise in our pursuit of the Sustainable Development Goals. Effective use of this expertise requires a strengthening of its epistemic foundations and a renewed focus on more practical policy problems.

The seventeen Sustainable Development Goals (SDGs) represent the United Nation’s aspirations for global development. Athough only one of them, goal 13, relates specifically to climate change, the strong synergies between climate and health, food, water, energy and social systems, mean that climate change will affect our progress towards almost every one^1^. Climate science and climate economics can help us navigate a route towards these goals, but the approaches that currently dominate in these disciplines limit their ability to contribute effectively and to provide the best possible guidance. The next decade needs to see a massive scale-up of research effort but not one invested in doing more of the same. Radical changes are needed. Breakthroughs will initially come not by focusing on the potential of exascale computing^2^, which provides a billion billion calculations per second and thereby facilitates higher resolution climate models, but rather from investing in integrated multi-disciplinary expertise. Two issues must be addressed. First is a better understanding and characterisation of the fundamental conceptual challenges in making climate projections. Second is the integration of multi-disciplinary knowledge and perspectives to provide the most robust information currently possible on specific questions of practical relevance to decision-makers and society. To support the SDGs the next decade requires investment in skills and expertise which bring together subjects as diverse as, for instance, stochastic and physical processes, philosophy of science, economics and water management. Computing and data remain essential but must play supporting roles rather than the lead positions they have ascended to over recent decades.

Observed and expected global warming stands on solid epistemic foundations, as does the expectation of increasingly severe impacts on, for instance, ecosystems, food systems, and water availability^3^. The existence of the threat is not in question^2^ and is accepted by the vast majority of scientists^4^. Consequently climate change science now concerns itself substantially with the details of the expected changes, such as how warming will be distributed regionally^5^, local changes in precipitation, heatwaves, and wildfires^6^, and probability distributions for parameters such as climate sensitivity^7^. The question for climate science going forward, one might say, is how to increase the spatial and probabilistic resolution of its forecasts.

One response has been to call for international collaboration and investment in exascale computing for climate modelling^2^; similar to CERN’s provision of particle acceleration in particle physics^8^. Currently Global Climate Model (GCM) outputs are critical for economic planning in relation to the SDGs because they are widely interpreted as a source of information regarding how different regions will experience different climatic changes; information which if reliable would be valuable in designing sustainable responses. However, questions about the reliability of GCM outputs for this purpose have been raised over a number of years^2,9–11^. Most recently Palmer and Stevens^2^ have discussed the inadequacies of current models in terms of the scale of global and regional biases which are often greater than the signals they aim to simulate. The presumption that these inadequacies can be remedied with better computers is best understood within a paradigm that assumes that the existence of reliable multi-decadal predictive models is currently prevented primarily by limited computational capacity. This assumption, however, is one which is rarely stated and even less often defended. Probabilistic predictions of low dimensional nonlinear systems can show high sensitivity to model formulation^12^ known as the hawkmoth effect, akin to the butterfly effect but related to model structure rather than initial values. Climate models may or may not show high sensitivity to the finest details of model structure but regional responses can certainly change substantially as model parameters are varied^9^. How close to reality then do climate models need to be to generate robust, reliable predictions of the spatial and probabilistic details of future climate change? How big a computer, how complex a model, and how high a resolution is necessary? Given that both the butterfly effect and the hawkmoth effect may affect the results, how big an ensemble do we need and how should it be designed? These fundamental questions have yet to be addressed.

The fact is that we do not know what the binding constraints are to achieving the desired predictive capability. Before ploughing billions into developing specialised computers and associated computer models^2^, it would be wise to first develop a good theoretical understanding of what is necessary and sufficient to build models capable of such high-resolution predictions.

Without such understanding, model-based predictions are likely to be over-quantified and over-constrained, underestimating the true uncertainty. This introduces two significant risks. First is the risk that such predictions encourage policy makers trying to make progress towards the SDGs to lock in inappropriate long-term investments; for instance when new flood protection infrastructure turns out to be either unnecessary or insufficient. Second is the risk of undermining the credibility of the larger research enterprise. If overly constrained projections turn out to be wrong, or simply replaced by very different projections from the next generation of models, it might encourage scepticism over the reliability of climate science more broadly. One response to these concerns is to acknowledge that because our uncertainty estimates are often founded on a limited set of models and observations we should not expect well-quantified probability distributions but rather seek less precise domains of possible outcomes^9^. Research aimed at exploring such domains using perturbed-physics ensembles could prioritise the wide exploration of uncertainty^9^. Such an approach arguably provides better information for use in planning sustainable responses and supporting activities to achieve the SDGs because it is less susceptible to the underestimation of uncertainty. Indeed in the short term it may very well lead to increasing estimates of uncertainty as wider ranges of model parameters are explored and a proliferation of equally credible model formulations are found. This would nevertheless represent valuable progress as it would present a more robust evaluation of what may be in store. Fixating too much on reducing estimates of uncertainty can undermine efforts to achieve more robust information.

Climate change economics has long been characterised by a parallel “race-for-resolution”. Climate economics has its origins in the project of constructing integrated assessment models to produce cost-benefit analyses of mitigation policies, and subsequent generations of these models provide more regional and sectoral disaggregation^13–15^. Weitzman^16^, however, has pointed out that under some mild epistemic constraints which would give rise to a fat-tailed probability distribution for the equilibrium climate sensitivity, the benefits of mitigation do not converge under the standard assumptions of cost-benefit analysis. Climate economists it seemed had spent decades attempting to provide ever-better numerical estimates of a benefit-cost ratio that could well be infinite. Even if the equilibrium climate sensitivity isn’t strictly fat-tailed, the benefit-cost ratio appears to be highly sensitive to the shape of the probability distributions for physical parameters which suffer from deep uncertainty^17^. Similarly now, an effort is underway to leverage vast amounts of historical weather and economic data to derive more precise and more disaggregated mappings between climatic conditions and economic damages^18^, but it appears that these types of inferences rest on some rather strong and implausible assumptions^19^. It would perhaps be wise to put a greater emphasis on trying to understand the fundamental limitations on these projects from the beginning. For instance, to what extent are historical data likely to contain information regarding long-term future damages resulting from climate change? Similarly, when uncertainty in one element of the problem overwhelms all other aspects is there a risk that complicated analysis could obscure rather than illuminate the main message? If our quantitative answers hinge on assumptions that are not well founded (e.g. the shape of the climate sensitivity distribution) then perhaps we should seek a better way of posing the questions.

One approach receiving increasing attention in the physical sciences is the plausible storyline concept^20^. This aims to provide detailed regional or local information about future changes in climate, conditioned on a set of plausible, clearly-presented assumptions but with no attempt to assess relative probabilities. By foregrounding the assumptions the approach has the flexibility to capture a wider range of possible futures and facilitate debate over their relevance. It has been discussed in general terms in a number of articles^10,20,21^ and applied in several specific cases^22–24^. It has substantial potential to support the SDGs by exploring interconnected physical and social storylines^25^, constrained by physical and social science understanding. Computer models may provide details^20,23–25^ but model simulations are ideally constructed to inform the storyline rather than being its foundation. In one example physical storylines were generated regarding the variety of ways in which the Indian summer monsoon might respond to climate change^22^, with these changes then linked to a variety of options for responding to water demand in southern India supported by simulations with a water resources model^25^.

There would also be significant value in adopting more multi-disciplinary and expertise-centred approaches. Anthropogenic climate change is taking the Earth along a never before experienced trajectory, towards a never before experienced state. Because empirical validation of our models is necessarily carried out within a different, probably very different, state of the system, confidence in statements about the future can only come from an understanding of the underlying processes at work; an understanding that goes beyond our models. We need to develop understanding of the fundamental dependencies and uncertainties inherent when trying to project the future under climate change. Much could be learned from simpler systems to help us develop the most informative experimental designs^26^. We should draw upon expertise from a range of disciplines to build an integrated picture that reflects our best understanding of the geographical, sectoral, physical, and temporal aspects of the climate change problem. Climate science research needs to be more guided by the questions addressed in the social sciences and economics while climate economics needs to be more aware of the sources of deep uncertainty in the response of the physical system. In the next decade we need to see an integration of expertise from a range of mathematical, physical, and social science disciplines in order to generate robust actionable information to help us plan for the future. Confidence arises from a foundation of expertise; data and models are essential tools, but neither they nor their outputs are the end goal.

How, then, do we achieve the rich possibilities of science and economics to support individual, national and international aspirations such as those represented by the SDGs? A major international effort is certainly needed but it must come with a change of focus. We need experts with understanding which spans physics and economics. Climate science and economics should obviously continue to search for answers to societally pressing questions, but they must be more sensitive to the epistemic constraints they face as a result of the limited conceptual foundations for extrapolatory projections. There is indeed a need to invest in a CERN-like research enterprise, but its goal should be to build integrated expertise, not just models.
